# Exploiting superspace to clarify vacancy and Al/Si ordering in mullite

**DOI:** 10.1107/S2052252518007467

**Published:** 2018-06-22

**Authors:** Paul Benjamin Klar, Iñigo Etxebarria, Gotzon Madariaga

**Affiliations:** a Departamento de Física de la Materia Condensada, Facultad de Ciencia y Tecnología, Universidad del País Vasco UPV/EHU, Apartado 644, Bilbao 48080, Spain; b Departamento de Física Aplicada II, Facultad de Ciencia y Tecnología, Universidad del País Vasco UPV/EHU, Apartado 644, Bilbao 48080, Spain

**Keywords:** Al/Si ordering, vacancy ordering, ceramics, superspace, incommensurate structures

## Abstract

Synchrotron single-crystal X-ray diffraction has revealed diffuse scattering alongside sharp satellite reflections for different samples of mullite (Al_4+2*x*_Si_2−2*x*_O_10−*x*_). Structural models have been developed in (3+1)-dimensional superspace that account for vacancy ordering and Al/Si ordering based on harmonic modulation functions. A constraint scheme is presented which explains the crystal-chemical relationships between the split sites of the average structure. The modulation amplitudes of the refinements differ significantly by a factor of ∼3, which is explained in terms of different degrees of ordering, *i.e.* vacancies follow the same ordering principle in all samples but to different extents. A new approach is applied for the first time to determine Al/Si ordering by combining density functional theory with the modulated volumes of the tetrahedra. The presence of Si–Si diclusters indicates that the mineral classification of mullite needs to be reviewed. A description of the crystal structure of mullite must consider both the chemical composition and the degree of ordering. This is of particular importance for applications such as advanced ceramics, because the physical properties depend on the intrinsic structure of mullite.

## Abstract 2

New superspace models with different modulation amplitudes indicate that any degree of ordering, from disordered to ordered, can be observed in mullite.

## 1. Introduction   

**Figure 1:**
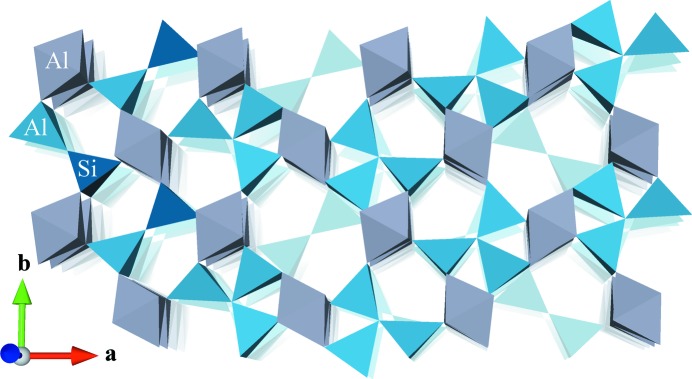
A model of the main structural elements of mullite. Chains of AlO_6_ octahedra along the *c* axis are interconnected *via* a network of dicluster and tricluster tetrahedra, which also form chains along the *c* axis. The Al/Si ratio determines the concentration of vacancies which in turn determines the concentration of triclusters, as each vacancy has a characteristic environment of two triclusters. Subsequent *AB* layers are shown with decreasing opacity to indicate that the vacancies do not form channels but are completely enclosed by the network of tetrahedra. In this model, below each vacancy there is a dicluster that consists of two Si cations.

The crystal structure of mullite (Al_4+2*x*_Si_2−2*x*_O_10−*x*_) has been a matter of investigation since the structure of the mineral sillimanite was solved (Taylor, 1928 ▸), which is chemically and structurally closely related to mullite. The main difference is the presence of oxygen vacancies with a concentration *x* that ranges between about 0.20 and 0.57, though it can be extended to about 0.9 depending on the synthesis conditions (Schneider *et al.*, 2015 ▸). The composition is often expressed as the oxide ratio Al_2_O_3_:SiO_2_, for example 3:2 for the 3/2-mullite (*x* = 0.25) or 2:1 for the 2/1-mullite (*x* = 0.4). Fig. 1 ▸ shows a polyhedral model of mullite with the characteristic triclusters around the oxygen vacancies. In particular, the distribution of these vacancies within the crystal structure is still a matter of debate. Furthermore, little is known about the distribution of Al and Si on the tetrahedral sites, though in general it is assumed that Si does not occupy tricluster tetrahedra, derived from the electrostatic bond-valence rule.

In reciprocal space, apart from the main Bragg reflections, a characteristic diffuse scattering pattern can be observed, which has been explained by correlated disorder of oxygen vacancies (Welberry & Butler, 1996 ▸; Freimann & Rahman, 2001 ▸, and references therein). Maxima in the diffuse scattering visible in the *hk*½ section were identified as satellite reflections with a modulation wavevector **q** = (α0γ), which depends on the chemical composition (Cameron, 1977 ▸). The dependence of α on the vacancy concentration *x* is different from the dependence of γ. The latter is ½ for 3/2-, 2/1- and 5/2-mullite, but for higher *x* a lowering of the symmetry to monoclinic is observed as γ deviates slightly from ½. The turnover vacancy concentration is not known, but it must be close to *x* ≃ 0.5. α decreases approximately linearly with increasing *x* in the orthorhombic case and seems to be constant in the monoclinic case (Ylä-Jääski & Nissen, 1983 ▸).

Agrell & Smith (1960 ▸) revealed that mullite shows either diffuse satellite reflections in *hk*½ sections alongside diffuse scattering, or sharp satellite reflection spots without diffuse scattering. A new terminology was suggested to distinguish between ‘D-mullite’ and ‘S-mullite’, which refer to samples with either diffuse or sharp satellite reflections, respectively. For more aluminous mullites, several electron diffraction studies revealed sharp satellite reflections of fifth and higher orders (Nakajima & Ribbe, 1981 ▸; Ylä-Jääski & Nissen, 1983 ▸). The observations suggested that vacancies in mullite with diffuse features are disordered, and mullites with sharp satellite reflections are ordered. A superspace model was developed that describes mullite as a mostly disordered structure with a weak occupational modulation, implying a low degree of vacancy ordering (Birkenstock *et al.*, 2015 ▸). In contrast, crystal structure models with an ordered distribution of vacancies were suggested earlier (Saalfeld, 1979 ▸; Ylä-Jääski & Nissen, 1983 ▸; Kahn-Harari *et al.*, 1991 ▸; Klar *et al.*, 2017 ▸), but only the most recent of these models was refined against diffraction data, which did not show any signs of diffuse scattering but sharp first-order satellite reflections. The refinement used different scale factors for the main and satellite reflections, assuming that the crystal consists of a component with complete vacancy ordering within a dis­ordered polymorph, and the ordered superspace model only accounts for the ordered component. The degree of ordering and the meaning of the model will be discussed in more detail in Sections 4.1 and 4.2.

The superspace approach was developed in the 1970s and established an elegant way to describe structures without lattice periodicity in a higher dimensional space, through which lattice periodicity is restored, and the observed three-dimensional reciprocal space is an aperiodic subspace of superspace. This approach was successfully applied to investigate and describe the modulated crystal structures of different classes of material, including minerals and pharmaceuticals (Bindi, 2008 ▸; Wagner & Schönleber, 2009 ▸). A description in superspace often marked the essential breakthrough in understanding a crystal structure which could not be understood before (Elcoro *et al.*, 2000 ▸; Izaola *et al.*, 2007 ▸; Pinheiro & Abakumov, 2015 ▸). It is therefore surprising that it was not applied to mullite in the late 1980s or 1990s, when the tools to understand the symmetry of modulated structures were already established. In Section 4.1 it will be shown that a meaningful model can be derived from a basic analysis of the superspace symmetry of mullite without a refinement.

In the present study, the crystal structures of several samples are investigated using synchrotron radiation. A disordered superspace model is developed and explained from a crystal-chemical perspective. Similarities to and differences from the model of Birkenstock *et al.* (2015 ▸) are pointed out and a new approach is used to analyse Al/Si ordering in superspace. The results allow us to understand and define the different degrees of ordering in mullite.

## 2. Experimental   

### 2.1. Samples   

Samples SA1, SA2 and SA3 originate from a batch of commercial aluminium silicate (Sigma–Aldrich), SA1 being the same crystal that was used for the work by Klar *et al.* (2017 ▸). From the previous study it was known that the composition of these samples corresponds to a vacancy concentration of about 0.4. In order to investigate a range of compositions, another sample labelled QG was prepared by mixing powders of γ-Al_2_O_3_ (Alfa Aesar) and amorphous SiO_2_ (Alfa) in a ratio of 5:2. After pressing the precursors into a pellet, the sample was kept in a flame until melting was observed and then quenched to room temperature. Crystallites that appeared single crystalline and transparent in a visible-light microscope were mounted on a polymer loop attached to a sample-holder pin.

### 2.2. Synchrotron single-crystal X-ray diffraction   

Measurements on SA1, SA2, SA3 and QG were carried out at the European Synchrotron Radiation Facility (ESRF) in Grenoble, France, on beamlines BM01 and ID28. The crystals were rotated about one axis (360° φ scan) and frames were recorded in 0.1° or 1.0° steps with a Dectris Pilatus 2M detector (BM01) or Pilatus 1M detector (ID28), respectively. The frames were treated with the *SNBL* toolbox (Dyadkin *et al.*, 2016 ▸) and the software *CrysAlisPro* (Rigaku Oxford Diffraction, 2017 ▸) was used for further data reduction. Details of the measurements and data reduction are given in Table 1 ▸.

## 3. Results   

### 3.1. Reciprocal-space analysis   

**Figure 2:**
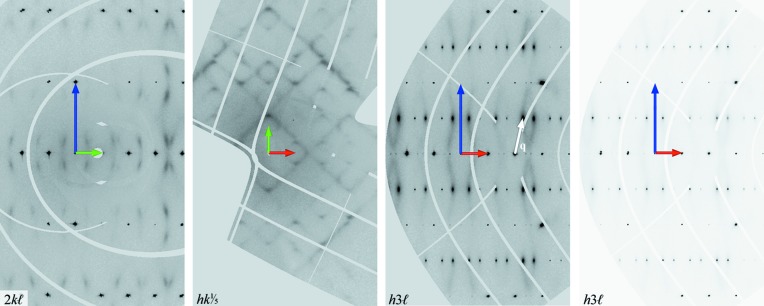
Portions of reciprocal space from the measurement of SA1 on ID28. The distorted grid of broad lines with zero intensity is due to the gaps between the detector modules. The reciprocal lattice vectors a*, b* and c* are shown as red, green and blue arrows, respectively, at the origin of each section. The *h*3*l* section is shown twice with two different greyscale settings (saturation limits are 4000 and 64 000 counts, respectively) to underline the sharpness of the satellite reflections on top of the diffuse discs. **q** is shown as a white arrow and indicates the similarity between the direction of **q** and the orientation of the diffuse streaks in sections perpendicular to b*.

The four measurements show the same features in reciprocal space, namely diffuse scattering and satellite reflections. The intensity of the diffuse scattering is weakly visible as a trace for the measurements on BM01 and is clearly visible in the reciprocal space of SA1 measured on ID28, due to the significantly more brilliant source. Reciprocal-space sections of that measurement therefore show the highest degree of detail and are used for subsequent analysis. First- and second-order satellite reflections were observed with a corresponding modulation wavevector **q** = [0.2978 (8), 0.0000 (11), 0.5000 (5)]. Besides the sharp satellite reflections, there are diffuse features in all reciprocal-space sections. However, the first-order satellite reflections are much more intense by about two orders of magnitude compared with the diffuse features and second-order satellites. The strongest diffuse features are elongated diffuse discs around strong satellite reflections. A second and a third set of satellite reflections are visible in sections perpendicular to a*, but these are not sharp and not clearly distinguishable from the diffuse scattering pattern in sections perpendicular to c*. This is shown in Fig. 2 ▸, where maxima are easily identified within the 2*kl* plane, but not in the 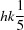 plane, where streaks form an intersecting diamond grid. Close to integer values of *l* the grid is rather continuous, but with increasing distance from integer values of *l* the streak pattern dissociates into separate features that transform continuously into the diffuse discs around the first-order satellites for *l* = *n* + ½. This indicates a direct relationship between the modulation and the diffuse scattering. In sections perpendicular to b* with integer values of *k*, weak streaks run from intense first-order satellite reflections towards second-order satellites, *i.e.* they run approximately parallel to **q** or *m_x_*:**q**, but wash out before they reach the second-order satellites (Fig. 2 ▸). Interestingly, there is no section in which diffuse streaks pass through any main reflection.

Reflection conditions are in agreement with the superspace group *Pbam*(*a*0½)0*ss*, as in former studies (Birkenstock *et al.*, 2015 ▸; Klar *et al.*, 2017 ▸); equivalent settings are discussed by Klar *et al.* (2017 ▸). This superspace group is used for all subsequent structure model refinements. Many main reflections show a clear splitting, suggesting that the sample is not perfectly single crystalline. In the case of SA1, the intensity ratio of the split reflections is about 4:1. However, this splitting is not observed for satellite reflections independent of the Bragg angle, which indicates that the satellites of the two grains exhibit different intensities relative to their main reflections. The measurements on BM01 show a clear splitting of the main and satellite reflections of SA2, whereas SA3 appears to be single crystalline. The main reflections of QG show partial splitting, but the main grain is strongly dominant and no splitting of satellite reflections is detected. The existence of few reflections with no clear relationship to the reciprocal lattice of mullite indicates the presence of an impurity phase. However, second-order satellite reflections could only be detected in reciprocal-space sections of SA1.

### 3.2. Refinement of the disordered superspace model in (3+1)-dimensional superspace   

The data set obtained on ID28 is not suitable for refinement because most of the main reflections are overexposed. In the following, the refinement of SA1 based on the measurement on BM01 is described. The refinement was carried out with the software *JANA2006* (Petříček *et al.*, 2014 ▸). A crucial aspect of the description of mullite in superspace is the atomic domain of O3, which is the bonding oxygen between two diclusters. As the site symmetry is 2/*m*, the symmetry restrictions require that all sine terms of the occupational modulation function of O3 are 0, and thus as a starting model the respective cosine term is set to an arbitrary value ≠ 0. A free refinement of the occupational modulation parameters of Al2 and Si2 was attempted, but the resulting modulation functions were not physically reasonable and different starting parameters led to different models without affecting the *R* factors. It is evident that the refinement is not sensitive to possible Al/Si ordering, and therefore the refinement parameters of Al2 and Si2 were constrained to be identical excluding the average occupancy. The ratio of the form factors of Si^4+^/Al^3+^ ranges between 1 and ∼1.16 and is ∼1.15 for sin(θ_max_)/λ of the measurements of this study, *i.e.* the reflections of the highest Bragg angles of this measurement are most sensitive to Al/Si ordering, though the contrast is still very low and apparently not sufficient to refine independent occupational modulation parameters for the respective atomic domains.

The overall composition is forced to be stoichiometric by applying a set of constraints on the occupancies *s* of Al2, Si2, Al3 and O4 as a function of the refinement parameter *s*_O3_, which is related to the vacancy concentration *x* = 2/3(1 − *s*_O3_). First-order harmonics for the occupational, displacive and anisotropic displacement parameter (ADP) modulation functions were refined. 40 correlations with a coefficient >0.9 between the modulation parameters of O3 and O4 and negative ADP tensors for some superspace coordinates indicate that the model requires more constraints. By setting the *U*_12_ modulation parameters of O3 and O4 to 0, only three strong correlations are observed and all parameters converge to physically reasonable values. Although the estimated standard uncertainties of many ADP modulation parameters are rather high, the fact that the ADP modulation does not show any conspicuous behaviour is taken as an indication that the electron density is correctly modelled.

**Figure 3:**
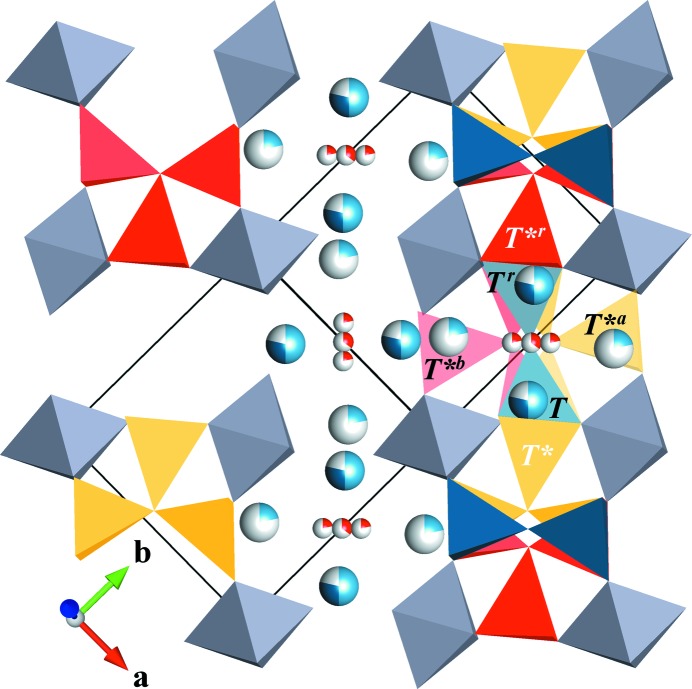
An average structure model of mullite with split sites and its decomposition into diclusters and triclusters. Unit-cell borders are marked by black lines. The fractional occupancy of each site is indicated by the filling of the sphere that represents the atom. A vacancy requires that all cation sites with black labels and the central oxygen sites are simultaneously vacant, and that two triclusters are present next to it, as shown on the left-hand side of the model.

Clear relationships between the occupational modulation functions (OMFs) of Al2/Si2, Al3, O3 and O4 emerge during the refinement. The refinement is stable without further constraints, but the estimated standard uncertainties are greatly reduced by applying constraints on the OMFs, which are derived below. Fig. 3 ▸ shows the unit cell of the average structure and the interpretation of the split sites in terms of the presence of diclusters, triclusters and vacancies. A detailed description of the sites and their labels is given in Table 2 ▸. Split *T* and T* sites are modulated in an antiphase relationship to avoid face-sharing tetrahedra. As the *T* site consists of the atomic domains of Al2 and Si2, their OMF amplitude is half that of Al3 on the T* site. In turn, Al3 and O4 must be modulated in phase and with the same amplitude. The red, blue and yellow clusters (Fig. 3 ▸) share the same *T* sites, *i.e.* the sum of the OMFs of the oxygen split sites (O3, O4*^a^* and O4*^b^*) must be the same as the sum of the OMFs of Al2 and Si2 to avoid dangling bonds. Hence the OMF of O3 can be expressed as OMF(O3) = OMF(Al2) + OMF(Si2) − OMF(O4*^a^*) − OMF(O4*^b^*). The constraints for first-order harmonics are summarized in Table 3 ▸ and the general form for *n*th-order harmonics is given in the supporting information (Table S2). Even without the constraints, the refinement fulfils the derived relationships within 1σ. The amplitude *A* of the OMF of Al2 is half that of Al3 [*A*_Al2_/*A*_Al3_ = 0.494 (8)]. As Al2 and Si2 are modulated identically, the overall *T* site has the same modulation amplitude as Al3 with a relative phase shift of ½.^1^ Al3 and O4 are modulated in phase with the same amplitude (*A*_Al3_/*A*_O4_ = 0.99 (8)]. From the equation for the OMF of O3, the amplitude ratio *A*_O4_/*A*_O3_ = |[1 − 2cos(απ)]^−1^| ≃ 5.5 is calculated, which agrees with the observed value of *A*_O4_/*A*_O3_ = 5 (3). The term 2cos(απ), with α being the first component of **q**, originates from the phase shift between the OMFs of O3 and its split sites O4*^a^* and O4*^b^*. Despite the large relative uncertainty in the amplitude of the OMF of O3, the refinement is in good agreement with the expected amplitude relationships. Applying the described constraint scheme increases *wR*(*F* ^2^) from 0.102 to 0.104, *i.e.* the model itself is not affected, but there is a significant improvement in the estimated standard uncertainties due to decreased correlations (Table S1). For this reason, the constrained model is preferred over free refinement. Selected atom-site parameters of the constrained refinement are given in Table 4 ▸ and the resulting model is described in the next section. The refinements from the other three measurements were carried out accordingly and relevant parameters are included in Table 4 ▸.

### 3.3. Description of the superspace model   

**Figure 4:**
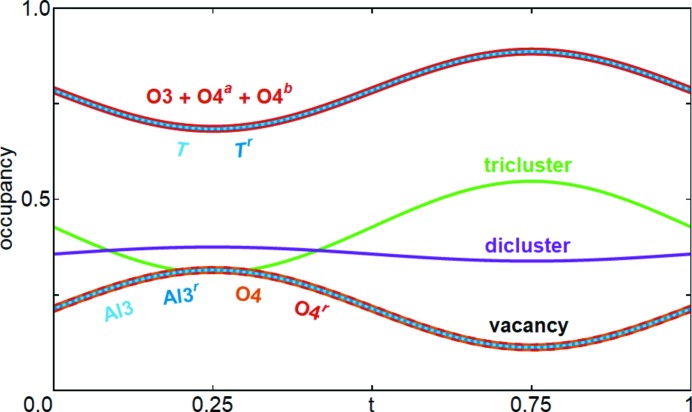
Occupational modulation functions of selected atomic domains. Site labels can be compared with Fig. 3 ▸. The curves of *T*, *T^r^* and (O3 + O4*^a^* + O4*^b^*) are identical. The same holds for the curves of Al3, Al3*^r^*, O4 and O4*^r^*, which are described in the text. The curves labelled tricluster, dicluster and vacancy sum up to a value of 1 and represent the respective fractions occupying the space around (0 ½ ½).

The refinements result in very similar superspace models and in the following the model of SA1 is described. Important differences between the refinements are pointed out at the end of this section. The site occupancy of O3 converged to 0.357 (7), which corresponds to a vacancy concentration of *x* = 0.428 (4). The occupational modulation as described in the previous section is based on the requirement that the occupancy of the cations on the *T* site is equal to the occupancy of the central O atoms, *i.e.* O3, O4*^a^* and O4*^b^*. The terms 1 − OMF(*T*) = 1 − OMF(*T^r^*) = 1 − OMF(O3) − OMF(O4*^a^*) − OMF(O4*^b^*) express the probability that a vacancy is present (Fig. 4 ▸). This probability is exactly the same as the occupancy of Al3, O4, Al3*^r^* and O4*^r^*, which form the tricluster environment of the vacancy. None of the OMFs reaches a value of 0 or 1, and thus diclusters, triclusters and vacancies occupy the space between octahedra with a probability that sums to 1. The probability composition for the clusters marked with black labels in Fig. 3 ▸ is included in Fig. 4 ▸. Although the occupancy merely describes a probability and the model does not require that two neighbouring sites with the same probability are simultaneously occupied, the strong correlation visible in Fig. 4 ▸ suggests that every vacancy is accompanied by two triclusters. This is also the only crystal-chemically reasonable interpretation which avoids dangling bonds and face-sharing tetrahedra. Studies of diffuse scattering confirm this tricluster environment and avoid other cluster assemblies like tetraclusters (Welberry & Butler, 1996 ▸).

**Figure 5:**
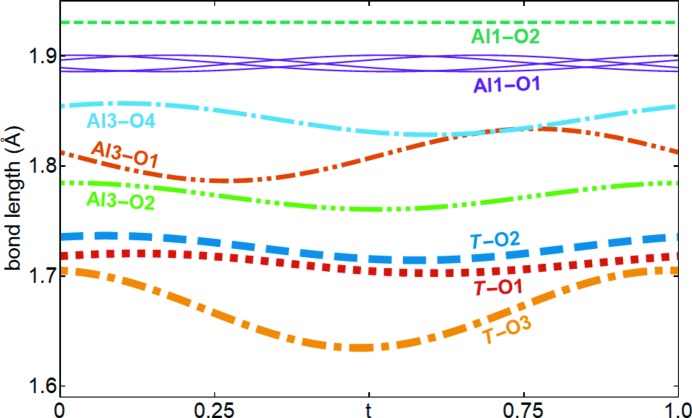
Modulated lengths of cation–oxygen bonds resulting from the displacive modulation.

Most atoms are displaced less than 0.02 Å due to the displacive modulation (Fig. S1). The largest displacement occurs for O1 within the *ab* plane by about 0.04 Å. Although Al1 and O2 are notably displaced out of the *ab* plane, the bond length is almost constant for any value of *t* (Fig. 5 ▸). The Al1—O1 distance does not vary much either and thus the volume of the octahedron changes by about ±0.001 Å^3^ due to modulation. The modulations of the bond lengths of *T* (*i.e.* Al2 and Si2) and Al3 are more pronounced. The Al3—O1 distance is correlated with the occupancy of Al3, *i.e.* O1 is closest to Al3 for *t* ≃ ¼, when the OMF of Al3 is at its maximum. This results in a distortion of the octahedron modulated by the presence of triclusters, diclusters and vacancies around the octahedron. The *T*—O3 bond length is probably related to Al/Si ordering on the *T* site.

As explained in Section 3.2, the OMFs of Al2 and Si2 could not be refined directly. Here, an approach is presented to derive these modulation functions from the volume of the tetrahedron. In the following, *V*_*T*,*d*_ refers to the average volume of the coordinating tetrahedron of the *T* site calculated from the coordinates of the average structure. As the *T* site is bonded to either O3, O4*^a^* or O4*^b^*, the value of *V*_*T*,*d*_ is the weighted sum of three volumes *V*_*T*,*d*,μ_ with a weighting factor that depends on the occupancy *s*_μ_ of the respective oxygen site. *V*_*T*,*r*_ refers to the volume calculated from the Al/Si ratio on that site as the sum of reference volumes 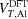 and 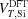, which are derived from a density functional theory (DFT) calculation, weighted by the occupancy of the cation
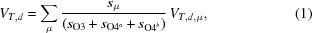


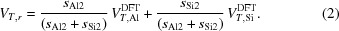
The calculation of *V*_*T*,*r*_ requires knowing the values of 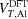 and 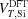, or alternative reference volumes. As no refinement of mullite is available without Al/Si disordered on the *T* site, the reference values were taken from a DFT study of an ordered model of 2/1-mullite (Klar, Aretxabaleta *et al.*, 2018 ▸), from which values of 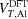 = 2.70 (5) Å^3^ and 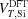 = 2.26 (4) Å^3^ were calculated as the averages of the volumes of ten independent AlO_4_ and six independent SiO_4_ tetrahedra, respectively.

**Figure 6:**
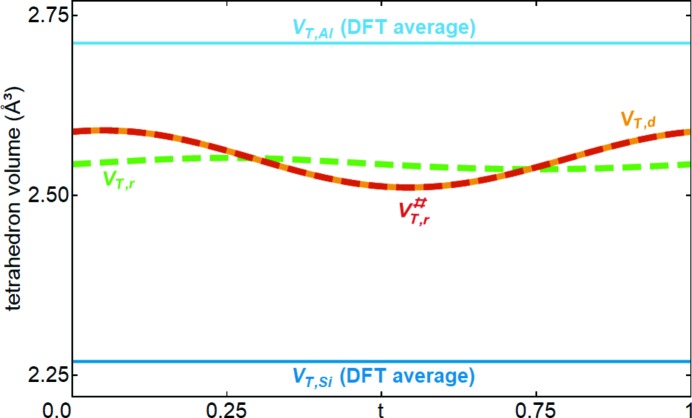
Modulation of the volume of the tetrahedral *T* site. *V*_*T*,*d*_(*t*) is the volume calculated using equation (1) from the modulated coordinates of the relevant oxygen atoms. *V*_*T*,*r*_(*t*) is calculated using equation (2). The discrepancy indicates that the initial occupational modulation functions of Al2 and Si2 are not in agreement with the observed variation of *V*_*T*,*d*_(*t*). Corrected modulation functions were calculated using equation (3) so that the expected volume 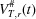 and observed volume *V*_*T*,*d*_(*t*) of the tetrahedron are consistent.

Using equations (1) and (2) and the defined reference volumes, for SA1 the calculated volumes are *V*_*T*,*d*_ = 2.5503 (19) Å^3^ and *V*_*T*,*r*_ = 2.54 (4) Å^3^, *i.e.* the observed volumes and the volumes expected from the composition are in good agreement. The slight discrepancy can be corrected by increasing 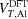 and 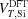 by one-sixth of the respective standard uncertainty. This correction is used in the following steps for convenience. If the displacive modulation and the occupational modulation are taken into account, *V*_*T*,*d*_ and *V*_*T*,*r*_ are functions of *t*. *V*_*T*,*d*_(*t*) varies between 2.511 and 2.590 Å^3^, and *V*_*T*,*r*_(*t*) between 2.536 and 2.552 Å^3^. Both curves are plotted in Fig. 6 ▸, which shows that *V*_*T*,*r*_(*t*) differs substantially from *V*_*T*,*d*_(*t*). This is not surprising, as during the refinement Al and Si were not distinguishable and thus the simplest model had to be used. However, new modulation functions 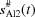 and 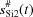 can be calculated from *V*_*T*,*d*_(*t*), so that 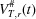 ≃ *V*_*T*,*d*_(*t*)
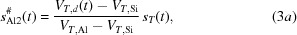


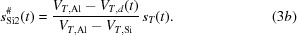

**Figure 7:**
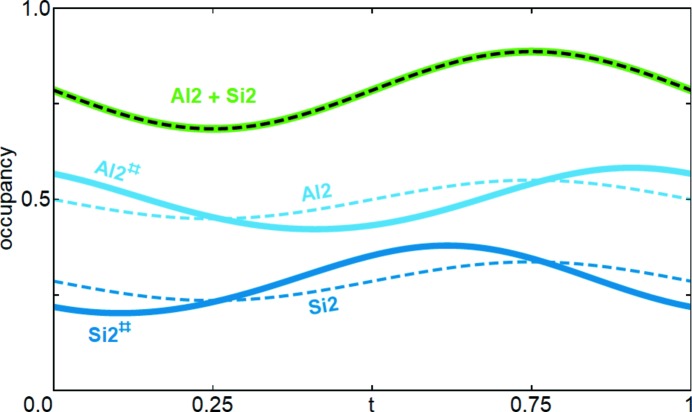
Occupational modulation of Al2 and Si2 from the constrained refinement (dashed lines) and from the functions derived from *V*_*T*,*d*_(*t*). The sum is the same in both cases (green/black curve).

*s_T_*(*t*) is the occupational modulation function of the *T* site, *i.e.* of Al2 + Si2, which is independent of Al/Si ordering. The corrected OMFs, labelled Al2^#^ and Si2^#^, are shown in Fig. 7 ▸. Using these modulation functions in the refinement results in a negligible improvement of *wR*(*F* ^2^) by about 0.001. The significant improvement is that the occupational modulation of Al2 and Si2 is now consistent with the observed volume of the tetrahedra, as 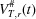 now overlaps with *V*_*T*,*d*_(*t*) in Fig. 6 ▸. The present approach and the resulting Al/Si ordering are discussed in Section 4.3.

The compositions of samples SA2, SA3 and QG were refined to *x* = 0.416 (4), 0.434 (6) and 0.426 (3), respectively. Elemental analyses of other studies of mullite report a higher uncertainty than the standard uncertainty estimated by the fitting algorithm, and therefore the uncertainties given here might be underestimated (Birkenstock *et al.*, 2015 ▸; Klar *et al.*, 2017 ▸). Nevertheless, the present samples all have a comparable composition close to the vacancy concentration of 2/1-mullite, including sample QG, for which the synthesis aimed for a vacancy concentration of 0.5. Apparently the quenching conditions were not appropriate for the synthesis of 5/2-mullite and a less aluminous mullite crystallized, with a composition that is favoured according to the phase diagram (Aksay *et al.*, 1991 ▸).

The deviations of the refined coordinates lie within the expected errors. Some ADPs, especially those of O3 and O4, differ more than other parameters, but this can be explained by the correlations between the parameters of O3 and O4 during the refinement and the sin(θ_max_)/λ of the measurements. The amplitude of the ADP modulation of the equivalent isotropic displacement parameter *U*_eq_ is within the 3σ range of *U*_eq_ in most cases. The similarity of the refined parameters of the basic structure confirms a consistent refinement.

The modulation functions show the same phase relationships, which in the case of the occupational modulation are fixed by the constraints. There is one remarkable difference between the refinements, which concerns the modulation amplitudes. A comparison shows that the ratio of the amplitudes of the OMFs of the different refinements is about 3.7:2.6:1:1.5 for SA1:SA2:SA3:QG. The corresponding ratios of the significant displacive modulation amplitudes are 2.9 (5):1.9 (3):1:1.2 (3) (only those amplitudes were considered where at least one of the function parameters was larger than 2σ). This significant difference is discussed in Section 4 in terms of different degrees of ordering.

The superspace model (SSM) published by Birkenstock *et al.* (2015 ▸) used a different constraint scheme, *i.e.* the atomic domains of Al2, Si2, Al3, O3 and O4 were set to have the same modulation amplitude. This is not reasonable, as Al2 and Si2 are on the same site, resulting in a modulation amplitude of the *T* site that is twice as large as the modulation amplitude of the other sites. Furthermore, O3 is modulated in phase with Al2 and Si2. This scheme suggests that a vacant O3 site is equivalent to the presence of a vacancy. This is not correct, because a vacancy requires that a total of seven sites are simultaneously vacant. The use of this constraint scheme leads to a strong ADP modulation of the O3 and O4 sites, with negative ADP tensors for some *t* sections and a strong displacive modulation of O4. The published data set of Birkenstock *et al.* (2015 ▸) was used for a refinement using the constraint scheme of Table 2 ▸. The ADP modulations improved, as well as the displacive modulation of O4, indicating that the constraints of the present study lead to a physically correct model. Interestingly, the *R* factors are hardly affected. The occupational modulation amplitude of O4 and Al3 is 0.0515 (6), which is in between those of SA2 and QG.

## 4. Discussion   

### 4.1. Comparison of ordered and disordered models   

The ordered superspace model presented by Klar *et al.* (2017 ▸) was based on the sample of SA1, but the resulting models seem to be very different. This section compares the models and points out their differences and similarities, starting with an analysis of the symmetry as both were refined in the superspace group *Pbam*(*a*0½)0*ss*.

**Figure 8:**
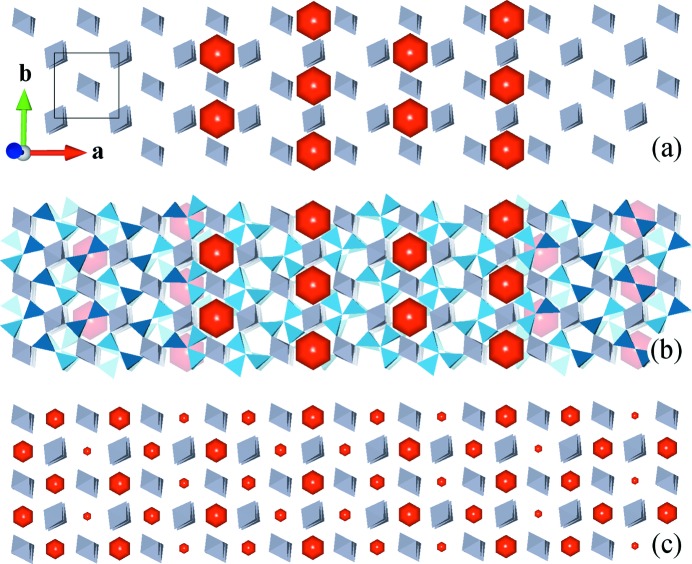
Comparison of the distribution of vacancies (red hexagons). (*a*) Vacancy distribution within one layer derived from the simplest model in superspace group *Pbam*(*a*0½)0*ss* with **q** = (0.3 0 0.5) and a vacancy concentration of 0.4 at coordinates with site symmetry 2/*m*, which is either vacant (red hexagon) or not vacant. This distribution can be derived without any knowledge of the average structure or chemistry of mullite. Average unit-cell borders (black) and octahedra (grey) are included for visual orientation. (*b*) Vacancy distribution with the structural model of the ordered superspace model. The subsequent layer, which is equal to the first layer shifted by 5**a**, is also shown with reduced opacity to indicate the relative location of vacancies in layers that are separated by 1**c**. (*c*) The hexagon size represents the probability that this site is vacant in the disordered SSM. In the case of SA1, this probability varies between 11.3% (smaller hexagons) and 31.5% (larger hexagons).

The vacancy distribution pattern can be derived as a function of the modulation wavevector **q** and the vacancy concentration *x* for a vacancy described by a block wavefunction centred at (0 ½ ½ 0) with site symmetry 2/*m*. The symmetry operators of the *a*- and *b*-glide planes establish a phase shift of ½ to a neighbouring vacancy site which is therefore centred at (½ 0 ½ ½). In a similar way, a phase shift of ½ is also established between two sites that are symmetrically related by *m*_*z*_. As a result, two vacancies in physical space are never connected by the vectors 〈½ ½ 0〉, 〈−½ ½ 0〉 or 〈001〉. Crystal-chemical considerations and models of disorder lead to the same basic avoidance pattern (Welberry & Butler, 1996 ▸), which is intrinsic to the symmetry of *Pbam*(*a*0½)0*ss*. The superspace group thus reserves space for triclusters around vacancies. These phase relationships also hold for harmonic modulation functions, and then an occupancy maximum of one site implies a minimum for the symmetrically related site. Fig. 8 ▸ depicts these relationships for the superspace group in general (Fig. 8 ▸ *a*), the ordered SSM (Fig. 8 ▸ *b*) and the dis­ordered SSM (Fig. 8 ▸ *c*).

The ordered SSM naturally exhibits the simple vacancy distribution pattern, resulting in two structural block units repeating throughout the structure (Fig. 8 ▸ *b*). In vacancy blocks, only vacancies and triclusters occur. In vacancy-free blocks, diclusters are present without any vacancies. This block pattern alternates along the *a* and *c* axes and is maintained throughout the structure.

The disordered SSM, based on first-order harmonic functions, resembles the patterns described by the ordered SSM, *i.e.* respective sites are likely to be vacant if there is a vacancy in the ordered SSM and a vacancy is unlikely to be present at sites where the ordered SSM prohibits a vacancy due to the above-described avoidance pattern. Thus, the vacancy distribution is approximately the superposition of the ordered SSM and an average statistical distribution of vacancies. Further aspects of the O3 domain are discussed in the supporting information in Section S4.

Due to this relationship, it is possible to use the same data sets as were used for the refinement of the disordered SSM for a refinement of the ordered SSM, although certain restrictions apply. (i) Main and satellite reflections require separate scale factors and only first-order satellite reflections may be used if only low-order satellites are present. (ii) Some parameters, especially the displacive and ADP modulation of shorter atomic domains like Al3, O3 and O4, are not refined due to instabilities or convergence to suspicious values. With these restrictions, the data sets of SA1, SA2, SA3 and QG give acceptable values for *wR*(*F* ^2^) of 0.138, 0.124, 0.149 and 0.113, respectively. The ratio of the scale factor of the satellite to that of the main reflection is a measure of the degree of ordering, like the modulation amplitude discussed in the last section. Comparing these ratios relative to the ratio of SA3 gives 3.53 (4):2.40 (3):1.000 (16):1.475 (17), which is almost equivalent to the relative proportions of the corresponding modulation amplitudes of the disordered SSM in Section 3.3.

The ordered model gives a straightforward interpretation of the origin of the modulation and of the dependence of the modulation on the composition, and a clear vacancy distribution pattern. Refinements with different scaling factors were interpreted in terms of ordered domains that are present within the mainly disordered mullite crystal (Klar *et al.*, 2017 ▸). However, there are many indications that the observed satellite reflections in the analysed samples are not caused by the presence of ordered domains. Structure factor calculations based on the DFT model used in Section 3.3 with a limit sin(θ_max_)/λ = 0.7 indicate that the mean 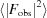 of reflections of different satellite order *m* are 1, 0.7, 0.5, 0.2 and 0.1 for *m* = 1, 2, 3, 4 and 5, respectively. Hence, higher-order satellites are slightly less intense than first-order satellite reflections. In the measurement of SA1, first- and second-order satellites are present and the ratio of the mean is 1:0.01. If the sharp satellite reflections in the measurement originate from ordered domains, much stronger second-order satellites must be present. Furthermore, higher orders with *m* > 2 are not observed at all, even though brilliant synchrotron radiation was used. The refinement of the disordered SSM gives very good *R* factors and the modulation parameters of all atomic domains are physically meaningful and realistic. In contrast, the refinement of the ordered SSM results in worse *R* factors and the ADP modulation and displacive modulation indicate problems with the model. Therefore, the disordered SSM is a better model of the electron density in superspace. Nevertheless, both models exhibit the same underlying vacancy ordering pattern with the same distribution of maxima in the 4*d* electron density, due to which the ordered model can be refined with the above-mentioned restrictions. Considering all these aspects, the ordered model is essential for understanding the vacancy ordering pattern in mullite, but the disordered model is the appropriate description of the crystal structure of mullite.

Precession electron diffraction tomography (PEDT) measurements on a broad range of samples with different compositions, including the commercial aluminium silicate sample, did not reveal ordered domains with high-order satellite reflections, indicating that the disordered SSM is also valid on a local scale. Details of these measurements will be published elsewhere (Klar, Palatinus & Madariaga, 2018 ▸).

### 4.2. Different degrees of ordering   

**Figure 9:**
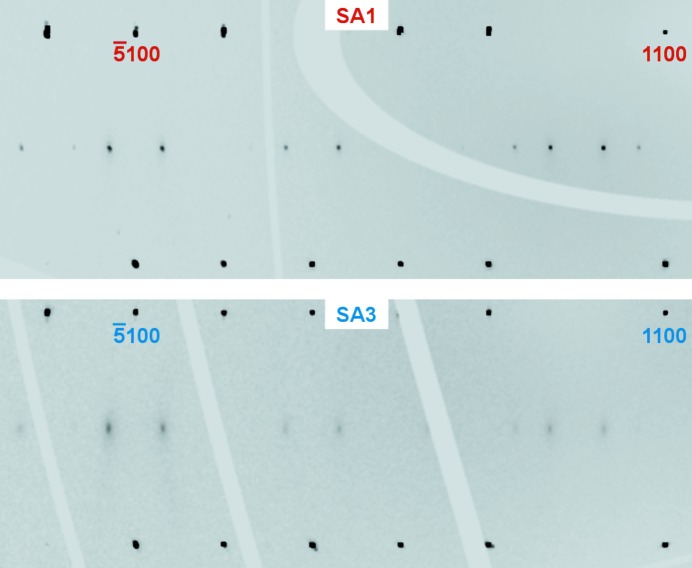
Parts of the *h*1*l* sections of SA1 and SA3, both from measurements on BM01, scaled for better comparison. Satellites of SA3 (bottom) appear weaker and more diffuse compared with SA1 (top) with sharp reflections.

According to the refined SSMs, the different modulation amplitudes are an intrinsic property of the samples. The modulation describes the long-range ordered deviation from the average structure with a periodicity defined by **q**. Correspondingly, the occupational modulation is the periodic increase and decrease of the occupancy with an amplitude that is larger in the case of SA1 and rather weak in the case of SA3. In other words, vacancies are more ordered in SA1 than in SA3. A direct comparison of the reciprocal-space sections of SA1 and SA3 supports this interpretation, because satellite reflections are clearly identified as reflections in SA1 but are weaker and more diffuse in the case of SA3 (Fig. 9 ▸). The presence of second-order satellites in the case of SA1 and their absence in all other samples also supports the result that SA1 is the most ordered sample of this study. Using the modulation amplitudes as a measure of the degree of ordering, QG is more ordered than SA3 and less ordered than SA2. The comparison of the refinements thus shows that mullite exists with different degrees of ordering.

Agrell & Smith (1960 ▸) reported on the existence of ‘S-mullite’ with sharp satellite reflections and without diffuse scattering, in contrast with ‘D-mullite’ with diffuse maxima as satellites and the characteristic diffuse scattering. The original samples, where S-mullite was the sample named Forster and D-mullite was sample number 58480 (Agrell & Smith, 1960 ▸; Cameron, 1977 ▸), were used in an electron diffraction study and, from their *h*0*l* sections, it seems that no second-order satellite reflections were present [*cf.* Figs. 3*c* and 3*d* in the work of Cameron (1977 ▸), compared with Fig. 9 ▸ of this work]. In a recent study, for which SA1 was measured with a laboratory diffractometer, diffuse scattering could not be detected (Klar *et al.*, 2017 ▸). In contrast, Fig. 2 ▸ clearly shows sharp satellite reflections alongside diffuse scattering and weak second-order satellites. Hence, mullite with low-order satellite reflections always exhibits diffuse scattering, but its visibility in especially small samples may require an X-ray source of higher brilliance, which is not commonly available in the laboratory. This also explains why Aramaki & Roy (1962 ▸) could not find ‘any intermediate layer lines’, even ‘in grossly overexposed photographs’ of a sample with sharp satellite reflections. The distinction into S-mullite and D-mullite should thus be avoided as they both exhibit diffuse features, and are both described by the same structural model with slightly different degrees of ordering.

All the refinements have in common that the occupancies are modulated within a small amplitude range. A physically meaningful model requires the occupancies to be modulated within a range between 0 and 1, which introduces limitations on the allowed amplitudes depending on the order of harmonics that are used to describe the modulation function. For example, the occupancy of the *T* site is 1 − *x*/2 and that of the O4 and T* sites is *x*/2. Therefore, the amplitude of the OMFs using only first-order harmonics is restricted to values ≤*x*/2. Note that all refinements discussed here are based on first-order harmonics and the amplitudes are smaller than *x*/2. A stronger modulation requires higher-order harmonics, which corresponds to higher-order satellite reflections. The respective constraints and hypothetical modulation functions of a more ordered mullite with third-order harmonics are presented in Section S3. In the literature, several electron diffraction studies give good examples of samples of different compositions and different orders of satellite reflections ranging from 1 to 7. The diffractograms of Cameron (1977 ▸) contain first- (Figs. 3*a*–3*d*), second- (Figs. 3*e*–3*f*) and fourth-order satellite reflections (Fig. 3*g*). Sayir & Farmer (1994 ▸) and Nakajima *et al.* (1975 ▸) reported on mullites with third-order satellite reflections. The selected-area electron diffraction pattern of Nakajima & Ribbe (1981 ▸) shows at least seventh-order satellite reflections for a mullite sample with monoclinic modulation wavevector **q**. Ylä-Jääski & Nissen (1983 ▸) reported on mullites with fifth-order satellite reflections and developed a fully ordered model, *i.e.* with occupancies of either 0 (unoccupied site) or 1 (occupied site), on the basis of images from high-resolution transmission electron microscopy (HRTEM), achieving a good agreement between recorded and simulated images. The vacancy distribution is in full agreement with the symmetry analysis of Section 4.1 and the HRTEM model can be decomposed into vacancy blocks and vacancy-free blocks. All results of the above-mentioned studies can be explained within the picture of a range of ordering introduced in this work, *i.e.* higher-order satellite reflections correspond to a higher degree of vacancy ordering. On this scale, the samples of this study are only slightly ordered.

The disordered SSM exhibits a periodicity of 1**b** and 2**c** and the structure is aperiodic along **a**, though commensurate models of 2/1-mullite can be approximated with a periodicity of 10**a**. However, the presence of diffuse scattering indicates that these periodicities are violated by correlated disorder. In reciprocal-space sections perpendicular to c*, the diffuse features are rather localized close to the satellites and expand to a pattern of diamond-shaped streaks in sections close to integer values of *l*. The vacancy distribution pattern of the disordered SSM, especially the periodicity of 2**c**, is likely to be preserved to a great extent, but along **a** and **b** it apparently deviates due to a variation in composition and ordering patterns. For a highly ordered sample ‘the pattern of diffuse streaks […] has not been observed in the diffraction experiments’ (Ylä-Jääski & Nissen, 1983 ▸), indicating that the diffuse scattering depends on the degree of ordering as well. Previous models that explained diffuse scattering did not consider different degrees of ordering (Welberry & Butler, 1996 ▸; Rahman *et al.*, 2001 ▸). More work is needed to better understand the diffuse scattering, which is currently under investigation.

### 4.3. Al/Si ordering   

**Figure 10:**
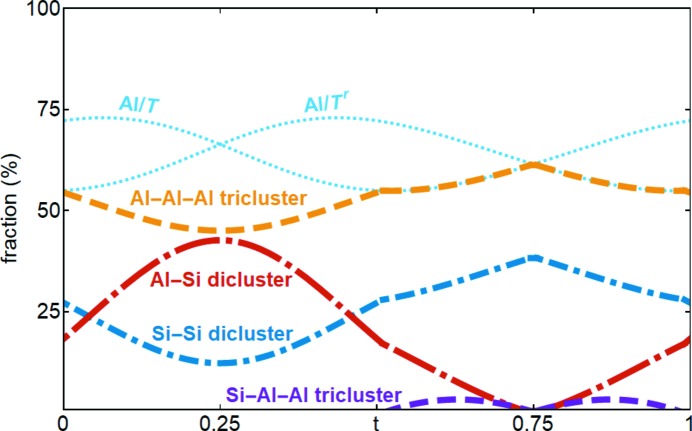
Al/Si ordering trends derived from the normalized occupancies of Al on the *T* and *T^r^* sites (light-blue dotted curves). For example, if the *T* site is occupied and *t* = 0.6, then it is either occupied by Al (56% probability) or Si (44% probability). The respective *T^r^* site is occupied by 69% Al and the *T*–*T^r^* pair in 59% of the cases is integrated in a tricluster, 56% of the Al–Al–Al type and 3% of the Si–Al–Al type. The remaining fraction form Al–Si and Si–Al diclusters (10% combined) and Si–Si diclusters (31%).

A free refinement of the OMFs of Al and Si on the *T* site failed, and therefore the respective modulation functions were derived from the modulation of the volume of the tetrahedron. Based on this adapted model, the Al/Si ordering can be analysed. First, the fraction of Al on the *T* sites is normalized with respect to the occupational modulation of the *T* site so that the Al fraction and the Si fraction sum up to 100%. Then, the fraction of *T* sites that are integrated in a tricluster is calculated. If Al–Al diclusters are avoided and Si is preferred in diclusters, the Al/Si ordering can be expressed as the occupancy of Si–Si diclusters, Al–Si diclusters and Al–Al–Al triclusters. For example, at *t* = 0.25, the *T* and *T^r^* site are both occupied by about 66% Al and 34% Si, and about 45% of the *T* sites are integrated in a tricluster. With the above-described assumptions, there is a unique solution for the fraction of *T*–*T^r^* pairs that form either Si–Si diclusters (13%), Al–Si diclusters (42%) or Al–Al–Al triclusters (45%). For ∼0.5 < *t* < ∼1, the tricluster fraction is higher than the Al fraction on one of the *T* sites, which requires the presence of an Si–Al–Al tricluster (Fig. 10 ▸). The presence of Si in triclusters was also identified by solid-state NMR measurements (King, 2014 ▸). The degree of ordering also affects the amplitudes of Al/Si ordering, so that for SA2, SA3 and QG the presence of Si–Al–Al triclusters is not necessary, but the general ordering pattern is the same for all samples. The phase shift of the modulation functions of subsequent diclusters in the chain of tetrahedra along the *c* axis is ½. Considering that vacancies are most likely to occur at *t* = ¼, a likely sequence is, for example, (vacancy, Si–Si, vacancy, …), (vacancy, Al–Al–Al, vacancy, …) or (Al–Si, Al–Al–Al, Al–Si, …). These results are in agreement with the DFT study of ordered mullite, which also confirms the presence of Si in triclusters (Klar, Aretxabaleta *et al.*, 2018 ▸).

The identification of Si–Si diclusters is relevant for the mineral classification of mullite, which for silicates is based on the type of network formed by SiO_4_ tetrahedra. In the current classification of Nickel–Strunz and Dana, the mineral mullite is classified as a nesosilicate with insular SiO_4_ units (Gaines *et al.*, 1997 ▸; Strunz & Nickel, 2001 ▸). This study shows, in agreement with DFT studies (Klar, Aretxabaleta *et al.*, 2018 ▸), that there are Si_2_O_7_ double tetrahedra groups alongside separate SiO_4_ tetrahedra, which makes mullite a sorosilicate with mixed SiO_4_ and Si_2_O_7_ groups. Therefore, we suggest that mullite be classified in Dana class 58 (instead of 52) and Nickel–Strunz class 09.BF (instead of 09.AF). Furthermore, in the classification of Dana, mullite is in the sillimanite subgroup 52.02.02a. Although there is a clear structural relationship with silli­manite, there is a similar relationship with andalusite, which in addition contains voids with a very similar geometry to the vacancy voids in mullite. Therefore, the classification in the sillimanite subgroup has an ambiguous character, which would be corrected by the suggested reclassification.

The problem of analysing Al/Si ordering on tetrahedral sites is common for many silicate families, and an equation similar to equation (3) was applied to derive the occupational modulation function from *T*—O bond lengths in feldspars (Angel *et al.*, 1990 ▸; Xu *et al.*, 2016 ▸). For both feldspar and mullite, reference values from pure SiO_4_ and AlO_4_ are not available from refinements, which is addressed by the approach of this work by deriving them from DFT models. With the refinement of SA1, the resulting curve of *V*_*T*,*r*_(*t*) from equation (2) is identical to the observed *V*_*T*,*d*_(*t*). However, the average occupancies must be checked carefully. For example, if *V*_*T*,Al_ and *V*_*T*,Si_ are taken from a refinement of sillimanite [Yang *et al.* (1997 ▸), *V*_*T*,Al_ = 2.782 (3) Å^3^ and *V*_*T*,Si_ = 2.192 (3) Å^3^], the resulting *s*_Al2_ = 0.477 (4), which deviates from the expected value of 0.5 by more than 5σ. This discrepancy is a measure of the quality of the reference volumes, and it is clear that the DFT calculation is a better source of these values. The advantage of the approach is that two different methods are combined, which must lead to a self-consistent result. The deviation from the expected occupancy is an immediate indicator to evaluate the result.

## 5. Summary and conclusions   

Synchrotron measurements of 2/1-mullite samples were used to investigate the vacancy and Al/Si ordering of the crystal structure. Diffuse scattering characteristic of mullite was observed, in combination with low-order satellite reflections. A new disordered superspace model was developed based on the refinement of four different samples with compositions close to that of 2/1-mullite. The relationships between the occupational modulation functions of the tetrahedral cations and the O3/O4 split sites are explained by crystal-chemical considerations and superspace symmetry. The refinements exhibit notably different amplitudes of the modulation functions concerning occupational and displacive modulations, which are discussed in the context of different degrees of ordering. Crystal structure analyses based on different scaling factors for main and satellite reflections allow a refinement of the ordered superspace model, with the interpretation that the sample is mostly disordered and few ordered domains are present in which vacancies are completely ordered (Klar *et al.*, 2017 ▸). Samples SA1, SA2 and SA3 originate from the same commercial batch, and their different degrees of ordering clearly show that the samples are not homogeneous. However, the absence of higher-order satellites and the observed intensity ratio of first-order to second-order satellites in the measurements of SA1 are not in agreement with the presence of a significant number of fully ordered domains. Therefore, the new disordered model of this work is an appropriate model to describe the crystal structure of mullite samples with low-order satellite reflections, and the degree of ordering is expressed by the amplitude of the occupational modulation functions. Many diffraction patterns in the literature exhibit different degrees of satellite reflections, indicating that mullite may adopt a broad range of degrees of ordering. The crystal-chemical relationships of the disordered model account for harmonic occupational modulation functions of any shape. Thus, the model of this study is the basis for describing mullite with any degree of ordering, and the stronger the ordering the more it resembles the ordered model, which represents the most ordered state.

DFT or molecular dynamics (MD) calculations are not possible with partially occupied atoms. Although the superspace symmetry of mullite allows the derivation of a simple vacancy-ordering distribution, recent MD studies use a random distribution of vacancies (Lacks *et al.*, 2005 ▸; Chen *et al.*, 2008 ▸; Zamani & Behdinan, 2017 ▸; Adabifiroozjaei *et al.*, 2018 ▸), and DFT calculations use a vacancy distribution that is constrained by size considerations but not by superspace symmetry considerations (Chen *et al.*, 2010 ▸; Aryal *et al.*, 2012 ▸). The different studies partly avoid Si on the T* site, but Al/Si ordering on the *T* site is not considered. The ordered model is a symmetry-consistent starting point for DFT calculations, which are limited to supercells with a few hundred atoms. The model is also suitable for MD simulations and disorder can easily be introduced if needed.

The results of a recent DFT study based on the ordered model were used in this study and proved to be useful to investigate the Al/Si ordering in superspace. A relationship between the presence of Si–Si diclusters and the presence of vacancies could be established from the analysis of the modulated volumes of the tetrahedra. The approach to deriving the Al/Si ordering on tetrahedral sites in combination with DFT calculations is, in principle, applicable to any silicate system. It leads to a consistent description of Al/Si ordering, the observed tetrahedra volumes and the composition constraints. The applicability of the reference volumes derived from DFT is easily assessed by the resulting average occupancy.

This work is consistent with, or explains discrepancies of, a number of former studies that applied different methods (Agrell & Smith, 1960 ▸; Cameron, 1977 ▸; Ylä-Jääski & Nissen, 1983 ▸; Kahn-Harari *et al.*, 1991 ▸; King, 2014 ▸; Klar *et al.*, 2017 ▸). In routine work on mullite using powder diffraction, satellite reflections are not observed and therefore not considered (Ren *et al.*, 2018 ▸; Figueiredo *et al.*, 2018 ▸; Lerdprom *et al.*, 2018 ▸; Ripin *et al.*, 2018 ▸; Sacks *et al.*, 1991 ▸). Sayir & Farmer (1994 ▸) reported on a strong variation in the tensile strength of different samples, with second-order satellites either present or absent, indicating that different degrees of ordering may, to some extent, account for the different properties. We therefore suggest the consideration not only of the composition and microstructure of mullite samples, but also of the degree of ordering, to investigate the relationship between the intrinsic structure and physical properties. An NMR study of sintered and fused mullite shows a visible difference between the respective ^29^Si NMR spectra (Schmücker *et al.*, 2005 ▸). Thus, NMR is a promising routine approach if a relationship between the degree of ordering and the NMR signal can be established. The superspace models of this work can help to deconvolute complicated NMR signals of mullite and relate them to superspace.

## Supplementary Material

Crystal structure: contains datablock(s) global, SA1, SA2, SA3, QG. DOI: 10.1107/S2052252518007467/gq5008sup1.cif

Structure factors: contains datablock(s) SA1. DOI: 10.1107/S2052252518007467/gq5008SA1sup2.hkl

Structure factors: contains datablock(s) SA2. DOI: 10.1107/S2052252518007467/gq5008SA2sup3.hkl

Structure factors: contains datablock(s) SA3. DOI: 10.1107/S2052252518007467/gq5008SA3sup4.hkl

Structure factors: contains datablock(s) QG. DOI: 10.1107/S2052252518007467/gq5008QGsup5.hkl

Additional discussion, tables and figures. DOI: 10.1107/S2052252518007467/gq5008sup6.pdf

13462ERV71a

CCDC references: 1843794, 1843795, 1843796, 1843797

## Figures and Tables

**Table 1:** Experimental details (*T* = 293 K)

	(I), SA1	(II), SA2	(III), SA3	(IV), QG
Crystal data				
Chemical formula	Al_4.856_Si_1.144_O_9.572 (4)_	Al_4.832_Si_1.168_O_9.584 (4)_	Al_4.868_Si_1.132_O_9.566 (6)_	Al_4.852_Si_1.148_O_9.574 (3)_
*M*_r_	316.3	316.5	316.2	316.3
Crystal system, space group	Orthorhombic, *Pbam*(*a*0½)0*ss*			
Wavevector **q**	0.2988 (9)a* + 0.5c*	0.301 (2)a* + 0.5c*	0.3068 (19)a* + 0.5c*	0.2948 (19)a* + 0.5c*
*a* (Å)	7.5787 (7)	7.577 (2)	7.5768 (13)	7.577 (2)
*b* (Å)	7.6707 (4)	7.6727 (18)	7.6760 (16)	7.6738 (19)
*c* (Å)	2.8836 (1)	2.8804 (10)	2.8833 (12)	2.8823 (10)
*V* (Å^3^)	167.64 (2)	167.46 (8)	167.69 (8)	167.59 (8)
*Z*	1	1	1	1
Radiation type	X-ray, λ = 0.7231 Å			
μ (mm^−1^)	1.08	1.08	1.08	1.08
Crystal size (mm)	0.06 × 0.05 × 0.03	0.07 × 0.05 × 0.03	0.05 × 0.05 × 0.02	Diameter < 0.01

Data collection				
Diffractometer	Four-circle diffractometer, ESRF beamline BM01			
Absorption correction	Absorption was corrected for by multi-scan methods. Empirical absorption correction using spherical harmonics, implemented in SCALE3 ABSPACK scaling algorithm (*CrysAlis Pro*)			
*T*_min_, *T*_max_	0.834, 1	0.761, 1	0.585, 1	0.569, 1
No. of measured, independent and observed reflections	3363, 678, 555	3079, 768, 544	3299, 667, 382	3576, 797, 454
*R*_int_	0.014	0.020	0.030	0.014
(sin θ/λ)_max_ (Å^−1^)	0.733	0.730	0.724	0.731
Range of *h*, *k*, *l*	*h* = −8→8	*h* = −10→10	*h* = −11→11	*h* = −11→11
	*k* = −11→11	*k* = −10→10	*k* = −10→10	*k* = −10→10
	*l* = −4→4	*l* = −4→4	*l* = −3→3	*l* = −4→4

Refinement				
*R*[*F* ^2^> 3σ(*F* ^2^)], *wR*(*F* ^2^), *S*	0.037, 0.104, 1.01	0.031, 0.089, 1.08	0.037, 0.111, 1.44	0.025, 0.097, 1.03
No. of reflections	678	768	667	797
No. of parameters	101	101	101	101
No. of constraints	33	33	33	33
Δρ_max_, Δρ_min_ (e Å^−3^)	0.37, −0.33	0.35, −0.39	0.43, −0.46	0.28, −0.30

Symmetry operations: (i) *x*_1_, *x*_2_, *x*_3_, *x*_4_; (ii) −*x*_1_, −*x*_2_, *x*_3_, *x*_3_ − *x*_4_ + ½; (iii) −*x*_1_ + ½, *x*_2_ + 1/2, −*x*_3_, −*x*_4_ + ½; (iv) *x*_1_ + ½, −*x*_2_ + ½, −*x*_3_, −*x*_3_ + *x*_4_; (v) −*x*_1_, −*x*_2_, −*x*_3_, −*x*_4_; (vi) *x*_1_, *x*_2_, −*x*_3_, −*x*_3_ + *x*_4_ + ½; (vii) *x*_1_ + ½, −*x*_2_ + ½, *x*_3_, *x*_4_ + ½; (viii) −*x*_1_ + ½, *x*_2_ + ½, *x*_3_, *x*_3_ − *x*_4_.

Computer programs: *CrysAlis Pro* (Version 1.171.38.46; Rigaku Oxford Diffraction, 2017 ▸) and *JANA2006* (Petříček *et al.*, 2014 ▸).

**Table 2:** Brief description of atom sites and selected symmetrically related sites

Label	Brief description
O1, O2	Four O1 and two O2 atoms form the octahedral coordination of Al1
O3	O3 is bonded to two corner-sharing tetrahedra labelled *T* and *T^r^*
O4	O4 is bonded to three tetrahedra, including the T* site
O4*^a^*, O4*^b^*	O4*^a^* and O4*^b^* are the split sites of O3. O4*^a^* forms a tricluster with *T*, *T^r^* and T*^a^ (pale-yellow tricluster in Fig. 3 ▸), and O4*^b^* with *T*, *T^r^* and T*^b^ (pale-red tricluster in Fig. 3 ▸)
Al1	Atom at the origin of the unit cell bonded to six oxygen atoms
Al2/*T*, Si2/*T*	Al2 and Si2 occupy the tetrahedral *T* site. The cation is always bonded to one O1 and two O2 atoms. The fourth oxygen is either O3, O4*^a^* or O4*^b^*
*T^r^*	*T* and *T^r^* form diclusters bonded by O3, or triclusters bonded by O4*^a^* or O4*^b^*
Al3/T*, Al3*^r^*/T*^r^	T* is part of a tricluster with O4 as bonding oxygen. The triclusters of T* and T*^r^ accompany a vacancy at O3/O4*^a^*/O4*^b^* (see Fig. 3 ▸)

Symmetry operations that relate the site of the asymmetric unit with the listed site: (*a*) *x*_1_ − ½, −*x*_2_ + , *x*_3_, *x*_4_ + ½; (*b*) −*x*_1_ + ½, *x*_2_ + ½, *x*_3_, *x*_3_ − *x*_4_; (*r*) −*x*_1_, 1−*x*_2_, *x*_3_, *x*_3_ − *x*_4_ + ½.

**Table 3:** Constraints scheme applied during the refinements α is the first component of the modulation wavevector **q**. The term cos(απ) originates from the phase shift between O3 and its split sites O4*^a^* and O4*^b^*.

Site	Occupancy *s*	Amplitude relative to O3	Phase shift relative to O3 in *t* space
O3	Refinement parameter	1	0
O4	(1 − *s*_O3_)/3	[1 − 2cos(απ)]^−1^	½
Al2	½	[2 − 4cos(απ)]^−1^	0
Si2	½ − *s*_Al3_	[2 − 4cos(απ)]^−1^	0
Al3	(1 − *s*_O3_)/3	[1 − 2cos(απ)]^−1^	½

**Table 4:** Selected parameters of the models from the constrained refinements Only parameters of atomic domains with occupancy *s* < 1 are shown. *A* is the amplitude of the modulation function of the respective parameter calculated from the cosine and sine components. *U*_eq_ is the equivalent isotropic displacement parameter.

Sample	SA1	SA2	SA3	QG
Vacancy concentration	0.428 (4)	0.416 (4)	0.434 (6)	0.426 (3)
Al2/Si2				
*x*	0.14896 (9)	0.14885 (5)	0.14905 (5)	0.14908 (4)
*x A*	0.00140 (3)	0.00092 (2)	0.00050 (4)	0.00058 (3)
*y*	0.33959 (6)	0.33985 (4)	0.34005 (5)	0.33987 (4)
*y A*	0.00152 (3)	0.00097 (2)	0.00048 (2)	0.00066 (3)
*U*_eq_ (Å^2^)	0.0121 (3)	0.00933 (17)	0.0096 (3)	0.01046 (17)
*U*_eq_ *A* (Å^2^)	0.00083 (6)	0.00060 (4)	0.00043 (7)	0.00043 (7)
*s*_Al2_	0.5	0.5	0.5	0.5
*s*_Si2_	0.286 (2)	0.2920 (19)	0.283 (3)	0.2868 (17)
*s A*	0.0506 (5)	0.0355 (3)	0.01345 (19)	0.02048 (19)
Al3				
*x*	0.2630 (4)	0.2626 (2)	0.2624 (2)	0.26226 (19)
*x A*	0.00119 (18)	0.00090 (10)	0.00049 (17)	0.00060 (14)
*y*	0.2056 (3)	0.2043 (2)	0.2058 (2)	0.2054 (2)
*y A*	0.00103 (14)	0.00081 (9)	0.00039 (8)	0.00062 (12)
*U*_eq_ (Å^2^)	0.0112 (7)	0.0086 (4)	0.0105 (6)	0.0121 (4)
*U*_eq_ *A* (Å^2^)	0.0026 (4)	0.00010 (20)	0.0025 (3)	0.0022 (3)
*s*	0.214 (2)	0.2080 (19)	0.217 (3)	0.2132 (17)
*s A*	0.1012 (8)	0.0711 (5)	0.0270 (4)	0.0409 (5)
O3				
*x*	0	0	0	0
*x A*	0.0029 (5)	0.0017 (3)	0.0016 (5)	0.0007 (5)
*y*	0.5	0.5	0.5	0.5
*y A*	0.0034 (4)	0.0018 (3)	0.0013 (3)	0.0008 (5)
*U*_eq_ (Å^2^)	0.020 (3)	0.0196 (19)	0.017 (3)	0.021 (2)
*U*_eq_ *A* (Å^2^)	0.0017 (15)	0.0011 (12)	0.0001 (13)	0.0002 (18)
*s*	0.357 (7)	0.376 (6)	0.349 (8)	0.360 (5)
*s A*	0.01838 (19)	0.01212 (10)	0.00379 (7)	0.00826 (10)
O4				
*x*	0.449 (2)	0.4483 (9)	0.4534 (10)	0.4543 (9)
*x A*	0.0016 (10)	0.0015 (6)	0.0009 (7)	0.0014 (6)
*y*	0.0509 (12)	0.0466 (9)	0.0482 (9)	0.0499 (8)
*y A*	0.0006 (8)	0.0015 (5)	0.0008 (5)	0.0004 (7)
*U*_eq_ (Å^2^)	0.017 (3)	0.0114 (17)	0.0120 (19)	0.0124 (16)
*U*_eq_ *A* (Å^2^)	0.0016 (16)	0.0017 (8)	0.0022 (11)	0.0013 (15)
*s*	0.214 (2)	0.2080 (19)	0.217 (3)	0.2132 (17)
*s A*	0.1012 (8)	0.0711 (5)	0.0270 (4)	0.0409 (4)
*V*_*T*,*d*_ (Å^3^)	2.5503 (19)	2.5573 (18)	2.5418 (20)	2.5488 (18)
*V*_*T*,*d*_ *A* (Å^3^)	0.0397	0.0254	0.0142	0.0157
*V*_*T*,*r*_ (Å^3^)	2.54 (4)	2.54 (4)	2.54 (5)	2.54 (4)

Restrictions induced by symmetry for Al2, Al3, O4: site symmetry *m*, displacive modulation of *z* forbidden, *U*_13_ and *U*_23_ must be 0 and may not be modulated. For O3 (site symmetry 2/*m*), the same restrictions apply, with the addition of: sine component of occupational modulation must be 0, cosine components of displacive modulation must be 0, sine components of ADP modulation must be 0

## Footnotes

1 Note that in this study the term ‘phase shift’ always refers to the phase difference between two modulation functions in *t* space. The modulation functions are functions of 2π*x*_4_ with *x*_4_ = **q**·**x** + *t*. The physical-space section *t* is constant for a structure model in physical space, which represents one subspace of the superspace model. The analysis of crystal-chemical aspects of the model requires the consideration of all the different environments of a selected site, which is an infinite number in the case of incommensurate structures. Instead of analysing the crystal chemistry of all sites in one crystal, the crystal chemistry of that site in different subspaces, *i.e.* different values of *t*, gives the same overview. Similar values of *t* usually describe a similar environment, but in physical space they describe two sites that are not close to each other. Therefore, *t*-plots give an overview of the possible crystal-chemical environments, but the analysis of a specific situation requires us to look at the same value of *t*.

## References

[bb1] Adabifiroozjaei, E., Hart, J. N., Koshy, P., Mitchell, D. R. G. & Sorrell, C. C. (2018). *J. Am. Ceram. Soc.* **101**, 428–439.

[bb2] Agrell, S. O. & Smith, J. V. (1960). *J. Am. Ceram. Soc.* **43**, 69–78.

[bb3] Aksay, I. A., Dabbs, D. M. & Sarikaya, M. (1991). *J. Am. Ceram. Soc.* **74**, 2343–2358.

[bb4] Angel, R. J., Carpenter, M. A. & Finger, L. W. (1990). *Am. Mineral.* **75**, 150–162.

[bb5] Aramaki, S. & Roy, R. (1962). *J. Am. Ceram. Soc.* **45**, 229–242.

[bb6] Aryal, S., Rulis, P. & Ching, W.-Y. (2012). *J. Am. Ceram. Soc.* **95**, 2075–2088.

[bb7] Bindi, L. (2008). *R. Fis. Acc. Lincei*, **19**, 1–16.

[bb8] Birkenstock, J., Petříček, V., Pedersen, B., Schneider, H. & Fischer, R. X. (2015). *Acta Cryst.* B**71**, 358–368. 10.1107/S205252061500757X 26027012

[bb9] Cameron, W. E. (1977). *Am. Mineral.* **62**, 747–755.

[bb10] Chen, J.-C., Chen, C.-S., Schneider, H., Chou, C.-C. & Wei, W. J. (2008). *J. Eur. Ceram. Soc.* **28**, 345–351.

[bb11] Chen, C.-S., Chou, C.-C., Chang, S.-W., Fischer, R. X. & Schneider, H. (2010). *Am. Mineral.* **95**, 1617–1623.

[bb13] Dyadkin, V., Pattison, P., Dmitriev, V. & Chernyshov, D. (2016). *J. Synchrotron Rad.* **23**, 825–829. 10.1107/S1600577516002411 27140164

[bb14] Elcoro, L., Perez-Mato, J. M. & Withers, R. (2000). *Z. Kristallogr.* **215**, 727–739.

[bb15] Figueiredo, J. M. R., Fernandes, I. M. M., Silva, V. J., Neves, G. A., Ferreira, H. C. & Santana, L. N. L. (2018). *Cerâmica*, **64**, 10–19.

[bb16] Freimann, S. & Rahman, S. (2001). *J. Eur. Ceram. Soc.* **21**, 2453–2461.

[bb12] Gaines, R. V., Skinner, H. C. W., Foord, E. E., Mason, B. & Rosenzweig, A. (1997). *Dana’s New Mineralogy: The System of Mineralogy of James Dwight Dana and Edward Salisbury*, 8th ed. Chichester: Wiley.

[bb17] Izaola, Z., González, S., Elcoro, L., Perez-Mato, J. M., Madariaga, G. & García, A. (2007). *Acta Cryst.* B**63**, 693–702. 10.1107/S0108768107037275 17873438

[bb18] Kahn-Harari, A., Abolhassani, S., Michel, D., Mazerolles, L., Portier, R. & Perez-Ramirez, J. G. (1991). *J. Solid State Chem.* **90**, 234–248.

[bb19] King, S. P. (2014). PhD thesis, University of Warwick, United Kingdom.

[bb20] Klar, P. B., Aretxabaleta, X. M., Etxebarria, I. & Madariaga, G. (2018). 26th Annual Conference of the German Crystallographic Society, 5–8 March 2018, Essen, Germany, abstract No. S15-03.

[bb21] Klar, P. B., de la Pinta, N., Lopez, G. A., Etxebarria, I., Breczewski, T. & Madariaga, G. (2017). *Acta Cryst.* B**73**, 377–388. 10.1107/S2052520617001652 28572548

[bb22] Klar, P. B., Palatinus, L. & Madariaga, G. (2018). Manuscript in preparation.

[bb23] Lacks, D. J., Hildmann, B. & Schneider, H. (2005). *Phys. Rev. B*, **72**, 214305.

[bb24] Lerdprom, W., Bhowmik, A., Grasso, S., Zapata-Solvas, E., Jayaseelan, D. D., Reece, M. J. & Lee, W. E. (2018). *J. Am. Ceram. Soc.* **101**, 525–535.

[bb25] Nakajima, Y., Morimoto, N. & Watanabe, E. (1975). *Proc. Jpn Acad.* **51**, 173–178.

[bb26] Nakajima, Y. & Ribbe, P. H. (1981). *Am. Mineral.* **66**, 142–147.

[bb27] Petříček, V., Dušek, M. & Palatinus, L. (2014). *Z. Kristallogr.* **229**, 345–352.

[bb28] Pinheiro, C. B. & Abakumov, A. M. (2015). *IUCrJ*, **2**, 137–154. 10.1107/S2052252514023550 PMC4285887 25610634

[bb29] Rahman, S., Feustel, U. & Freimann, S. (2001). *J. Eur. Ceram. Soc.* **21**, 2471–2478.

[bb30] Ren, Q., Li, H., Wu, X., Huo, Z., Hai, O. & Lin, F. (2018). *Int. J. Appl. Ceram. Technol.* **15**, 554–562.

[bb31] Rigaku Oxford Diffraction (2017). *CrysAlis Pro*. Version 1.171.38.46. Rigaku Oxford Diffraction, Oxfordshire, United Kingdom.

[bb32] Ripin, A., Mohamed, F., Choo, T. F., Yusof, M. R., Hashim, S. & Ghoshal, S. K. (2018). *Radiat. Phys. Chem.* **144**, 63–68.

[bb33] Saalfeld, H. (1979). *Neues Jahrb. Miner. Abh.* **134**, 305–316.

[bb34] Sacks, M. D., Bozkurt, N. & Scheiffele, G. W. (1991). *J. Am. Ceram. Soc.* **74**, 2428–2437.

[bb35] Sayir, A. & Farmer, S. C. (1994). *MRS Proc.* **365**, 11–20.

[bb37] Schmücker, M., Schneider, H., MacKenzie, K. J. D., Smith, M. E. & Carroll, D. L. (2005). *J. Am. Ceram. Soc.* **88**, 2935–2937.

[bb38] Schneider, H., Fischer, R. X. & Schreuer, J. (2015). *J. Am. Ceram. Soc.* **98**, 2948–2967.

[bb39] Strunz, H. & Nickel, E. (2001). *Strunz Mineralogical Tables*. 9th ed. Stuttgart: Schweizerbart Science Publishers.

[bb40] Taylor, W. H. (1928). *Z. Kristallogr.* **68**, 503–521.

[bb41] Wagner, T. & Schönleber, A. (2009). *Acta Cryst.* B**65**, 249–268. 10.1107/S0108768109015614 19461136

[bb42] Welberry, T. R. & Butler, B. D. (1996). *J. Eur. Ceram. Soc.* **16**, 187–193.

[bb43] Xu, H., Jin, S. & Noll, B. C. (2016). *Acta Cryst.* B**72**, 904–915. 10.1107/S205252061601578X 27910841

[bb44] Yang, H., Hazen, R. M., Finger, L. W., Prewitt, C. T. & Downs, R. T. (1997). *Phys. Chem. Miner.* **25**, 39–47.

[bb45] Ylä-Jääski, J. & Nissen, H.-U. (1983). *Phys. Chem. Miner.* **10**, 47–54.

[bb46] Zamani, S. M. M. & Behdinan, K. (2017). *Ceram. Int.* **43**, 12239–12248.

