# Supplementary material for: Exploiting superspace to clarify vacancy and Al/Si ordering in mullite
Source: IUCrJ. 2018 Jun 22;5(Pt 4):497–509. doi: 10.1107/S2052252518007467 (PMC6038955; doi:10.1107/S2052252518007467)
Supplement: Supplementary file 6 [file m-05-00497-sup6.pdf]

IUCrJ

Volume 5 (2018)

Supporting information for article:

Exploiting superspace to clarify vacancy and Al/Si ordering in mullite

Paul Benjamin Klar, Iñigo Etxebarria and Gotzon Madariaga

S1. Refinement of SA1 with and without constraints on OMF

Table S1 compares the effect of the constraint scheme (Table 2) on the refinement. The respective data of Table 4 (refinement with constraints) are included for easier comparison with the results of the refinement without constraints on the OMFs.

Table S1 Comparison of atomic parameters from the refinements of SA1 with and without constraints on the OMF. Descriptions as in Table 4.

	with constraints	without constraints	with constraints	without constraints
	Al2	Al2	O3	O3
x	0.14896 (9)	0.14896 (9)	0	0
x A	0.00140 (3)	0.00141 (3)	0.0029 (5)	0.0029 (11)
y	0.33959 (6)	0.33959 (6)	0.5	0.5
y A	0.00152 (3)	0.00154 (2)	0.0034 (4)	0.0031 (16)
U_{eq} (Å^2)	0.0121 (3)	0.0121 (3)	0.020 (3)	0.020 (3)
U_{eq} *A* (Å^2)	0.00083 (6)	0.00062 (7)	0.0017 (15)	0.0020 (17)
s	0.5	0.5	0.357 (7)	0.358 (7)
s A	0.0506 (5)	0.0502 (6)	0.01838 (19)	0.020 (11)
	Al3	Al3	O4	O4
x	0.2630 (4)	0.2629 (4)	0.449 (2)	0.448 (2)
x A	0.00119 (18)	0.00120 (17)	0.0016 (10)	0.0016 (14)
y	0.2056 (3)	0.2057 (3)	0.0509 (12)	0.0504 (13)
y A	0.00103 (14)	0.00103 (14)	0.0006 (8)	0.0006 (8)
U_{eq} (Å^2)	0.0112 (7)	0.0111 (7)	0.017 (3)	0.016 (3)
U_{eq} *A* (Å^2)	0.0026 (4)	0.0024 (4)	0.0016 (16)	0.0007 (18)
s	0.214 (2)	0.214 (2)	0.214 (2)	0.214 (2)
s A	0.1012 (8)	0.1017 (11)	0.1012 (8)	0.103 (8)
$V_{T,d}$ (Å^3)	2.5503 (19)	2.5503 (18)		
$V_{T,d}$ *A* (Å^3)	0.0397	0.0395		
$V_{T,r}$ (Å^3)	2.54 (4)	2.54 (4)		

S2. Comparison of modulation amplitudes

In the following several *t*-plots allow to visually compare the absolute displacements and occupational modulation. Plots of SA1 refer to the refinement without the constraints of Table 2 and can be compared with the Figures of the main article. The plots of SA2, SA3 and QG are based on the constrained refinements. For better comparison, plots of the same category use the same scale.

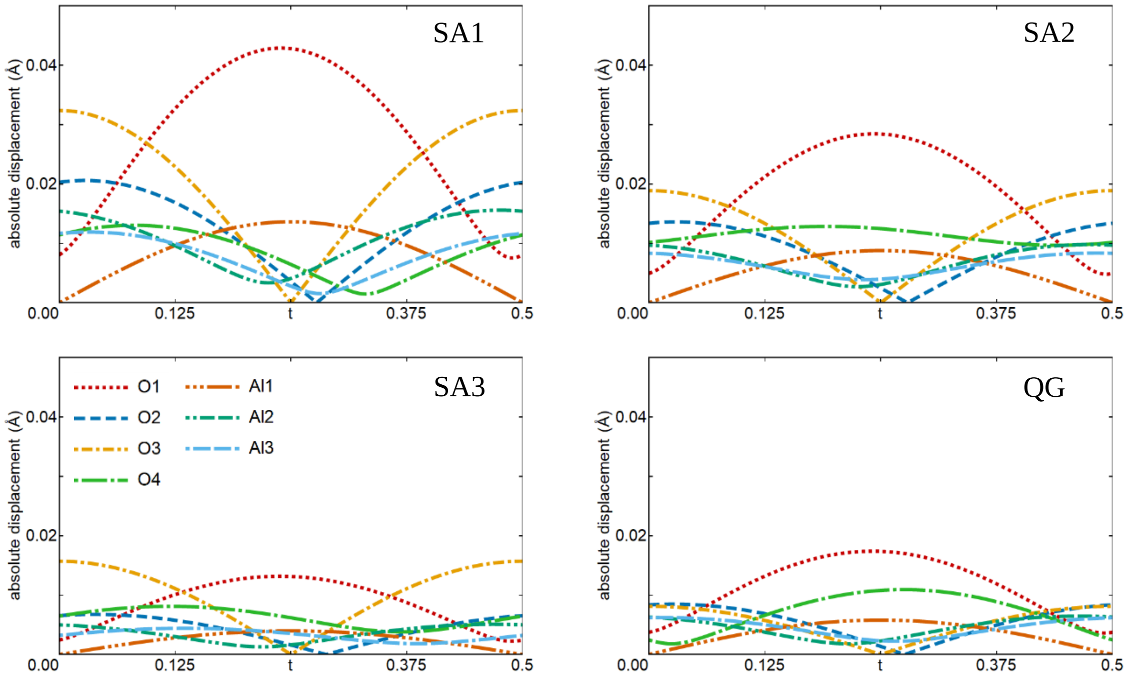

Figure S1 Absolute displacement of atoms from their average position due to the displacive modulation. Al1 and O2 are only displaced along the *c* direction, the other atoms within the *ab*-plane. The legend is shown in the plot of SA3.

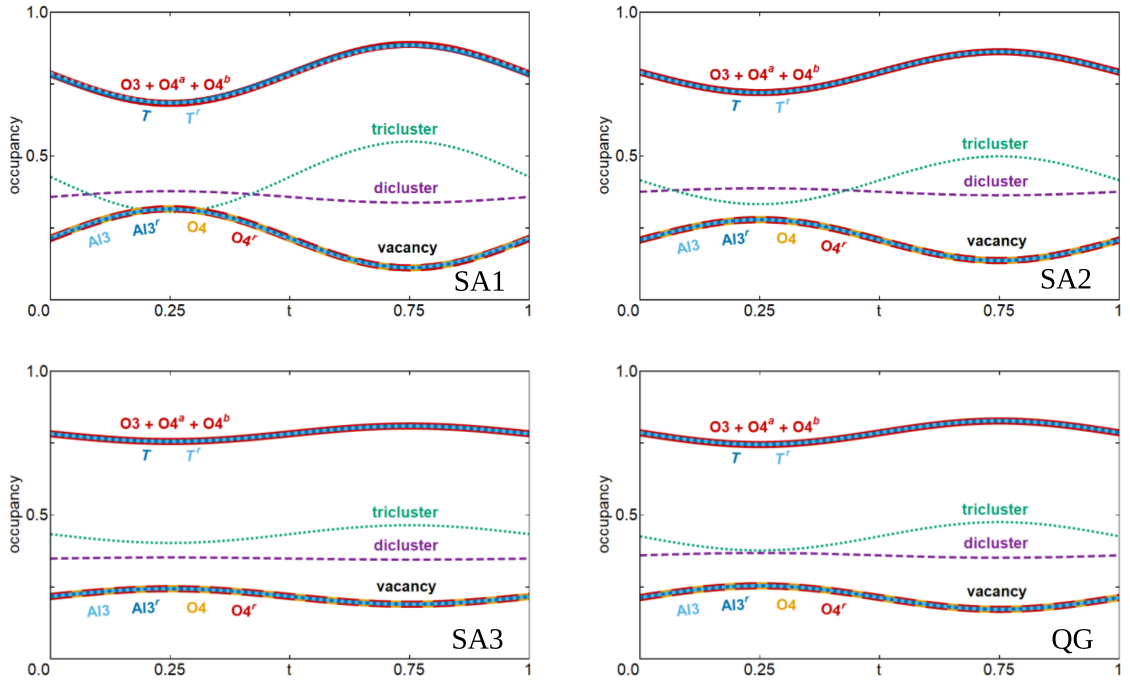

Figure S2 Comparison of occupational modulation functions (cf. Fig. 4)

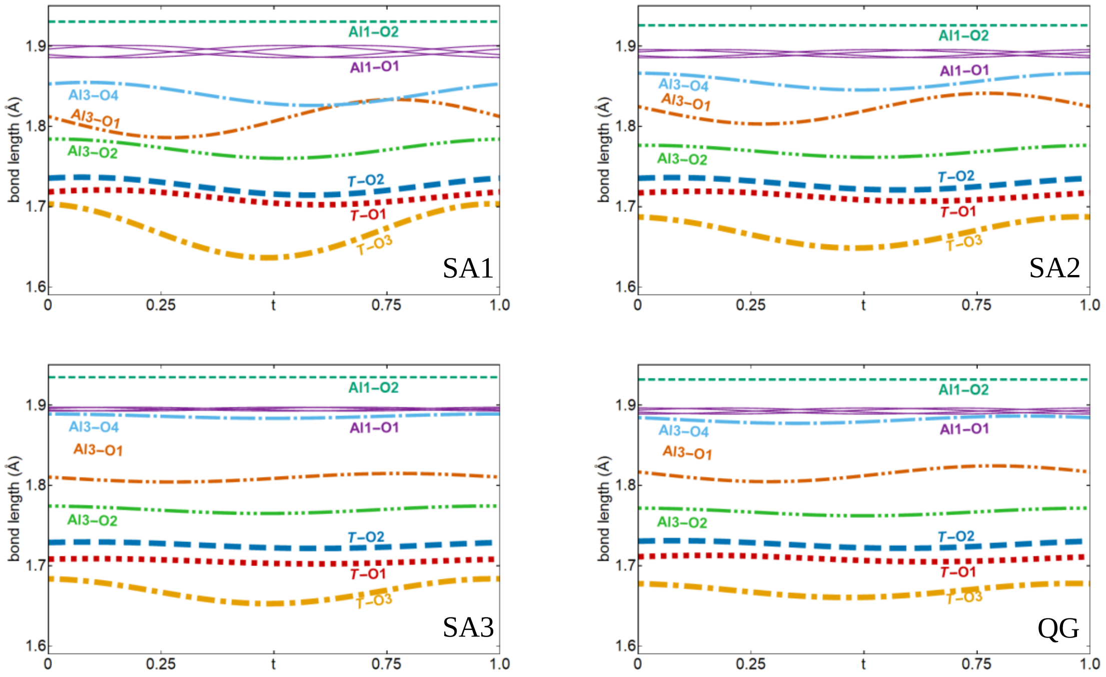

Figure S3 Comparison of bond lengths (cf. Fig. 5)

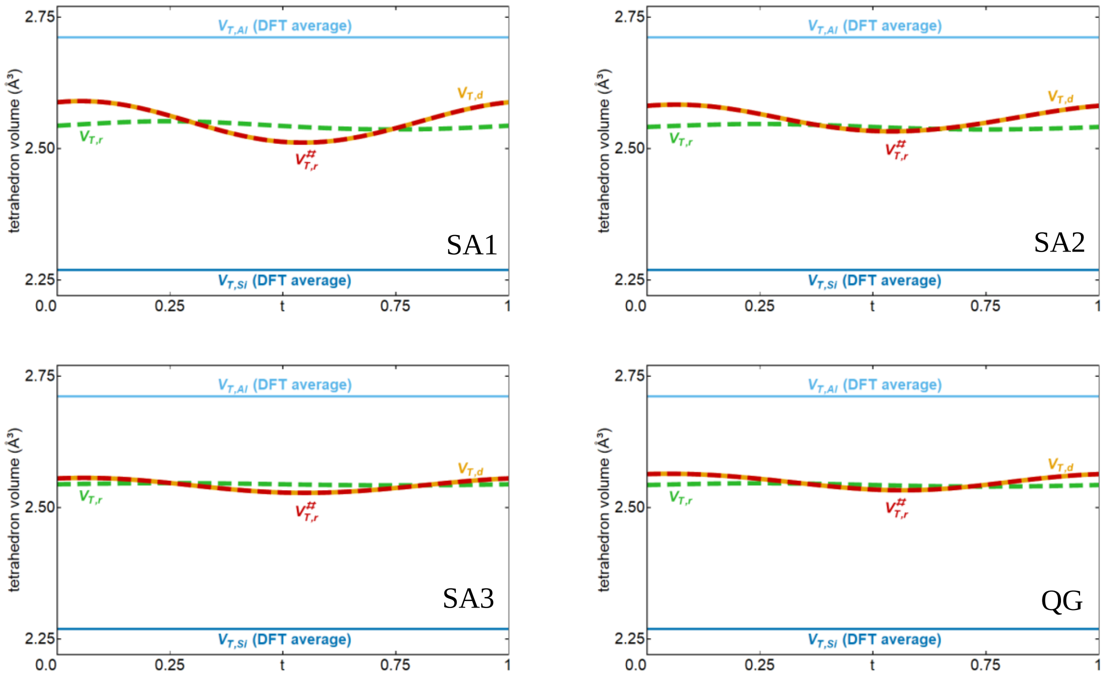

Figure S4 Comparison of the modulated volumes of the tetrahedral *T* site. (cf. Fig. 6)

S3. Mullite structures with higher degree of vacancy ordering

In section 4.1 the disordered SSM based on 1st order harmonics and the ordered SSM based on block wave functions are compared and discussed. The developed constraint scheme of section 3.2 holds for any degree of ordering and is given in Table S2 in a more general form (cf. Tab. 3) for harmonic modulation functions of order *n*. Figure S5 shows the occupational modulation functions (cf. Fig. 4) of a hypothetical 2/1-mullite with modulation amplitudes 0.25, −0.15 and 0.05 for 1st, 2nd and 3rd order harmonics of O4 and Al3. The respective occupational modulation amplitudes of the O3 domain are −0.044, −0.057 and 0.15.

Table S2 General form of constraints on the occupational modulation functions with n^{th} order harmonic functions optimized for a refinement of the occupation modulation function of O3.

Site	Amplitude factor of nth order harmonic	Factor for cosine component of nth order harmonic	Factor for sine component of nth order harmonic
O3	1	$\cos[n2\pi\alpha x_{O3}] = 1$	$\sin[n2\pi\alpha x_{O3}] = 0$
O4	$-(1+2\cos[n\pi(1+\alpha)])^{-1}$	$\cos[n2\pi\alpha x_{O4}]$	$\sin[n2\pi\alpha x_{O4}]$
T	$(1+2\cos[n\pi(1+\alpha)])^{-1}$	$\cos[n2\pi\alpha x_T]$	$\sin[n2\pi\alpha x_T]$
Al3	$-(1+2\cos[n\pi(1+\alpha)])^{-1}$	$\cos[n2\pi\alpha x_{Al3}]$	$\sin[n2\pi\alpha x_{Al3}]$

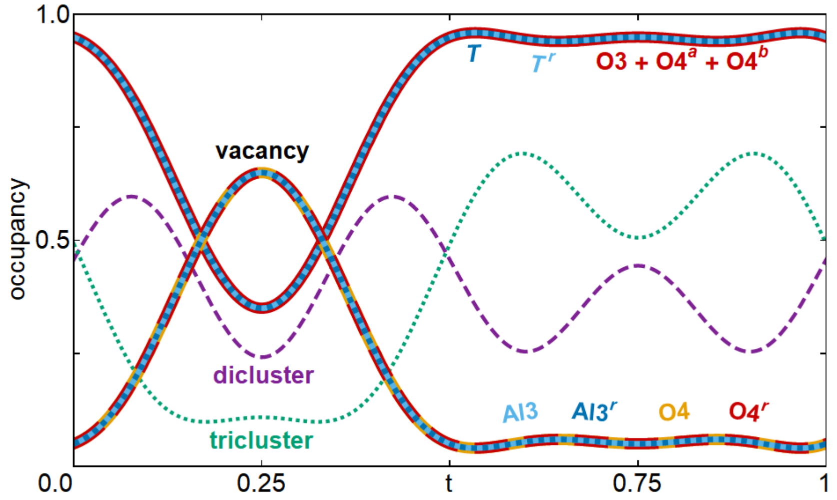

Figure S5 Occupational modulation functions of selected atomic domains of a hypothetical 2/1-mullite based on 3rd order harmonic functions. Labels are identical to Fig. 4.

S4. Shape of occupational modulation function of O3

In the refinements the OMF of O3 stands out in comparison to the other OMFs because the amplitude is notably lower. The function describes probability that a dicluster is present. In the ordered SSM, the atomic domain of O3 consists of three block wave functions of O3a, O3b and O3b^r. An analogous model can be incorporated into the disordered SSM by decomposing the OMF of O3 into three harmonic functions. These components cannot be determined through the refinement which is only sensitive to the superposition, but they can be derived from the ordered SSM assuming that the modulation amplitude of O3b is identical with Al3 and O4. This decomposition is shown in Figure S5. The resulting distribution of O3 is not affected by this alternative description, i.e. the occupancy of diclusters is only weakly modulated throughout the structure. However, a comparison with Figure 10 shows a clear correlation between O3a and the presence of Si-Si diclusters on the one hand, and between O3b/O3b^r and the presence of Al-Si diclusters on the other. This relationship was also identified in the DFT model (Klar, Aretxabaleta *et al.*, 2018).

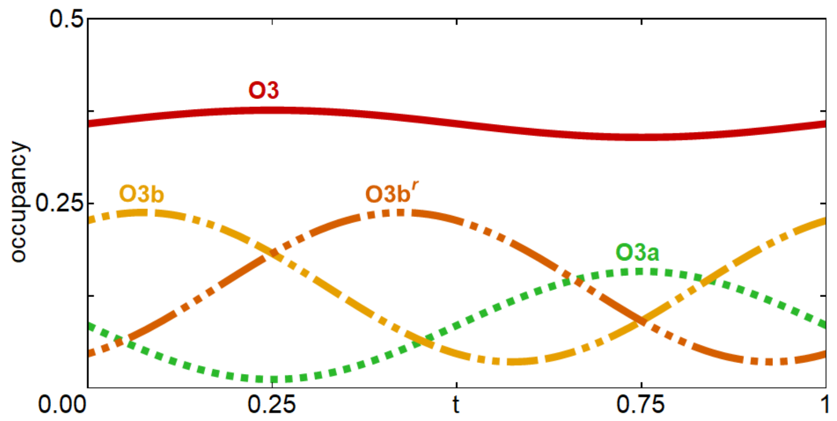

Figure S6 Possible decomposition of the O3 atomic domain into O3a, O3b and O3b′. The phase shift between O3a and O3b was derived from the constraints of the ordered SSM.
